# Prevalence and Symptom Characteristics of Irritable Bowel Syndrome Among Bronchial Asthma Patients in Pakistan

**DOI:** 10.7759/cureus.12231

**Published:** 2020-12-22

**Authors:** Parkash Bachani, Love Kumar, Naresh Kumar, Muhammad Khizar Memon, Sidra Memon, Sana Irfan, Owais Alam, Besham Kumar

**Affiliations:** 1 Internal Medicine, Liaquat University of Medical and Health Sciences, Jamshoro, PAK; 2 Internal Medicine, Jinnah Sindh Medical University, Karachi, PAK; 3 Internal Medicine, Jinnah Postgraduate Medical Centre, Karachi, PAK

**Keywords:** asthma, irritable bowel syndrome, rome criteria

## Abstract

Introduction: Irritable bowel syndrome (IBS) is a common gastrointestinal disorder that leads to a variety of symptoms including abdominal discomfort and change in stool frequency and consistency. Asthma is a common disease of the airway. Some studies have suggested that a relationship between IBS and asthma exist, while others have contradicted the claim. This study aims to determine the prevalence of IBS in asthmatic patients and compare their symptoms with symptoms of IBS patients in non-asthmatic patients.

Methodology: In this case-control study, 100 known and documented asthmatic patients were included as cases, and 100 non-asthmatic healthy patients were included as controls from July to August 2019. These patients were given a questionnaire based on ROME II criteria for the diagnosis of IBS. Prevalence and symptoms of IBS were compared between cases and controls. A probability level, *P* < 0.05 was considered significant.

Result: IBS was found in 41 out of 100 asthma patients (41%) and 18 out of 100 controls (18%) with a P-value of 0.0005 and was more common in females in both asthmatic (63.41%) and non-asthmatic patients (66.66%). Symptoms such as abdominal pain/distress (63.41% vs. 11.11%, P-value: 0.0013) and bloating (82.92% vs. 33.33%, P-value: 0.0005) were significantly higher in asthmatic patient with IBS compared to non-asthmatic patient with IBS.

Conclusion: Prevalence of IBS among asthma patients was significantly higher as compared to non-asthmatics. Routine screening of asthma patients and further studies to understand the pathogenesis underlying association between IBS and asthma should be conducted to detect and manage such patients effectively.

## Introduction

Irritable bowel syndrome (IBS) is a common gastrointestinal tract (GIT) disorder. Patients suffering from IBS show a variety of symptoms including changes in stool consistency and frequency, and pain in the abdomen. These symptoms catch medical attention, however, all the relevant investigations show normal results. These classical symptoms are reported by 15-20% of the general population [1]. Patients suffering from IBS show multiple gastrointestinal tracts and extra-intestinal signs and symptoms, leading to significant socioeconomic stress affecting their quality of life adversely [2]. Asthma is a chronic inflammatory disease of the airway characterized by increased responsiveness of the tracheobronchial tree to many stimuli. Like IBS, asthma is also known to reduce patients’ quality of life and is commonly associated with other common gastrointestinal disorders such as gastroesophageal reflux disease (GERD) and eosinophilic esophagitis [3,4]. 

Various studies have been conducted that have associated IBS with asthma [5-7]. Powell et al. reported an increased incidence of GIT symptoms in patients with allergic diseases like allergic rhinitis and asthma [8]. Studies have suggested an association between gastrointestinal system disorders and respiratory tract disorders, in such a way that asthma was reported more in patients already suffering from IBS [9]. On the contrary, other studies did not find any association between asthma and IBS, in patients after performing a methacholine challenge test [10,11].

There is no local study available to the best of our knowledge that studies the association between asthma and IBS in Pakistan. In this study, we aim to compare the prevalence and symptoms of IBS among asthmatic and non-asthmatic patients.

## Materials and methods

A total of 100 patients with a confirmed diagnosis of asthma were enrolled from the pulmonary clinic during the period from July to August 2019, in this case-control study. Patients with acute asthma exacerbation, history of organic bowel disease, diabetes, hypertension, or cardiac disease were excluded from the study. Patients who were taking medication other than for asthma were also excluded from the study. Gender and age-matched healthy controls (n = 100) were included in the study. Patient enrollment was started after appropriate approval was taken from the ethical review board.

All patients and healthy controls filled a questionnaire based on demographics and ROME II criteria, which is considered the gold standard for diagnosis of IBS [12]. According to ROME II, diagnostic criteria for IBS, the symptoms of IBS includes abdominal discomfort without any evidence of organic pathological process, require at least 12 weeks consecutive or non-consecutive, in the preceding 12 months with at least two of the following three features: (1) relieved with defecation, (2) change in the frequency of stool, and/or (3) change in form (appearance) of stool. The questionnaire was translated into the local language and was explained to participants as well. The individual symptoms of IBS were analyzed and recorded in the questionnaire. Physical examination was performed for all participants and no significant finding was identified in both case and control group. 

Statistical Package for Social Sciences (SPSS, version 23 Inc. Chicago, IL, USA) was used for data analysis. Prevalence and symptoms of IBS were compared between case and control to find any association and significant difference using chi-square. The odds ratio (OR) was calculated using an online calculator medical. A probability level, P < 0.05 meant that there is a significant difference between the two groups and the null hypothesis is void.

## Results

The mean age of participants in the asthmatic group was 25 ± 6 years and in the non-asthmatic group, it was 24 ± 6 years. Irritable bowel syndrome was found in 41 out of 100 asthma patients (41%) and 18 out of 100 controls (18%; P-value, 0.0005). The odds ratio was 3.16 (95% CI, 1.65-6.04) (Table 1).

**Table 1:** Irritable Bowel Syndrome in Asthmatic vs. Non-Asthmatic Patients

Irritable Bowel Syndrome	Asthmatic Patient (n=100)	Non-Asthmatic Patient (n=100)	Odds Ratio (95% Confidence Interval)	P-Value
Yes	41 (41%)	18 (18%)	3.16 (1.65-6.04)	0.0005
No	59 (59%)	82 (82%)		

Irritable bowel syndrome was more common in females in both asthmatic (63.41%) and non-asthmatic patients (66.66%) (Table 2).

**Table 2:** Comparison Between Asthmatic Patients With IBS vs. Non-Asthmatic Patients With IBS IBS: irritable bowel syndrome

Gender	Asthmatic Patient With IBS (n=41)	Non-Asthmatic Patient With IBS (n=18)
Male	15 (36.58%)	6 (33.33%)
Female	26 (63.41%)	12 (66.66%)

Abdominal pain/distress (63.41% vs. 11.11%; P-value, 0.0013) and bloating (82.92% vs. 33.33%; P-value, 0.0005) were significantly more common in asthmatic patient with irritable bowel syndrome compared to non-asthmatic patient with IBS (Table 3).

**Table 3:** Symptomatology of IBS Between Asthmatic and Non-Asthmatic Patients IBS: irritable bowel syndrome

IBS Symptoms	Asthmatic Patient With IBS (n=41)	Non-Asthmatic Patient With IBS (n=18)	Odds Ratio (95% Confidence Interval)	P-Value
Abdominal pain/distress	26 (63.41%)	2 (11.11%)	13.86 (2.79-68.78)	0.0013
Abdominal Pain relieved by passing stool	18 (43.90%)	6 (33.33%)	1.56 (0.49-4.98)	Not significant
Stool more than 3 per day	18 (43.90%)	10 (55.55%)	0.62 (0.20-1.91)	Not significant
Stool less than 3 per week	15 (36.58%)	7 (38.89%)	0.90 (0.28-2.83)	Not significant
Loose or watery stool	17 (41.46%)	7 (38.89%)	1.11 (0.35-3.45)	Not significant
Mucus in stool	9 (21.95%)	6 (33.33%)	0.56 (0.16-1.92)	Not significant
Bloating	34 (82.92%)	6 (33.33%)	9.71 (2.71-34.71)	0.0005

## Discussion

IBS manifests with many debilitating symptoms, including abdominal pain, bloating, muscle pain, and headache, and diagnosing it can be very challenging, considering the variability and fluctuation of disease symptoms over time and the overlap of symptoms with other conditions, such as lactose intolerance [2].

In our study, irritable bowel syndrome was found in 41% of asthmatic patients and 18% of non-asthmatic patients. This was comparable to a study conducted in Kuwait, which showed that 39.15% of asthmatic patients had IBS compared to 17.93% in non-asthmatic patients [7]. The odds ratio in this study 3.16 (1.65-6.04) was comparable to various other studies. Yilmaz et al. reported an odds ratio of 2.65 (95% CI, 1.25-5.57), meanwhile, Ozol et al. reported an odds ratio of 3.21 (95% CI, 1.53-6.70) in their study [5,6]. A meta-analysis showed a I_2_ value of 0% (P=0.8) and an OR of 2.6 (95%CI 1.9-3.5) [13]. In a study by Kennedy et al., they found that IBS, GERD, and symptomatic bronchial hyper-reactivity occur more frequently together than expected, and these conditions are independently associated with each other [14]. However, there are conflicting studies about the association of asthma and IBS as well and according to these studies, no significant association exists between the two [10,11].

In this study, abdominal pain/ distress and bloating were significantly more common in asthmatic patients with irritable bowel syndrome compared to non-asthmatic patients with IBS. This was comparable to a study in Kuwait, which also showed similar findings [7]. However, in the same study, non-asthmatic patients experienced increased stool frequency which was more than three per day, as compared to asthmatic patients, which is inconsistent with this study [7].

The association between IBS and asthma can be explained by the fact that they both have similar pathogenesis like dysregulation of contractility and tone of the smooth muscles, which can explain the presence of an association between the two [15,16]. An altered immune system is another proposed mechanism in the pathogenesis of IBS and asthma. Increased numbers of mast cells have been found in the gut of some IBS patients leading to altered muscle contraction and enteric nerve system imbalance in animals [6,17].

To the best of our knowledge, this is the first study in Pakistan that studies the prevalence and symptoms of IBS in asthmatic patients in the local population. However, there are few limitations as well. The study cannot establish a causal relation. It is a single centric study, so sample size diversity was reduced. Further large-scale studies are needed to understand the association of IBS in asthmatic patients.

## Conclusions

This study showed that a relationship between IBS and asthma exists. Abdominal discomfort and bloated feeling of the abdomen were more common in asthmatic patients. Because of the high prevalence of IBS in asthma, routine screening should be done for asthmatic patients. Moreover, large-scale studies must be conducted to confirm the association. Further studies to understand the pathogenesis underlying association between IBS and asthma should be conducted as well. 
